# Associations Among Suicidal Ideation, White Matter Integrity and Cognitive Deficit in First-Episode Schizophrenia

**DOI:** 10.3389/fpsyt.2018.00391

**Published:** 2018-08-28

**Authors:** Yicheng Long, Xuan Ouyang, Zhening Liu, Xudong Chen, Xinran Hu, Edwin Lee, Eric Y. H. Chen, Weidan Pu, Baoci Shan, Robert M. Rohrbaugh

**Affiliations:** ^1^Department of Psychiatry, Second Xiangya Hospital, Central South University, Changsha, China; ^2^Mental Health Institute, Second Xiangya Hospital, Central South University, Changsha, China; ^3^Hunan Key Laboratory of Psychiatry and Mental Health, Chinese National Clinical Research Center on Mental Health Disorders, National Technology Institute of Psychiatry, Changsha, China; ^4^Department of Psychiatry, University of Hong Kong, Hong Kong, Hong Kong; ^5^State Key Laboratory of Brain and Cognitive Sciences, University of Hong Kong, Hong Kong, Hong Kong; ^6^Medical Psychological Center, Second Xiangya Hospital, Central South University, Changsha, China; ^7^Key Laboratory of Nuclear Analysis, Institute of High Energy Physics, Chinese Academy of Sciences, Beijing, China; ^8^Department of Psychiatry, Yale University School of Medicine, New Haven, CT, United States

**Keywords:** first-episode schizophrenia, suicide, suicidal ideation, diffusion tensor imaging (DTI), white matter, cognitive function

## Abstract

**Objective:** The study was aimed to investigate the possible associations among suicidal ideation, brain white matter (WM) integrity and cognitive deficit in first-episode schizophrenia (FES) using diffusion tensor imaging.

**Methods:** The sample contained 18 FES patients with suicidal ideation (SI+), 45 FES patients without suicidal ideation (SI–) and 44 healthy controls. The Calgary Depression Scale for Schizophrenia was used to measure the suicidal ideation and depression symptoms. The whole brain WM integrity and three domains of cognitive function: working memory, verbal comprehension as well as processing speed were compared between the three groups.

**Results:** Compared with SI–, SI+ showed preserved WM integrity as indicated by significantly higher factional anisotropy (FA) or lower mean diffusivity (MD) in multiple WM tracts, and higher FA coupled with lower MD in bilateral posterior corona radiata. Compared with SI−, SI+ were more depressed and had less cognitive deficit in working memory and verbal comprehension. The fiber tracts in bilateral posterior corona radiata connect to the precuneus as shown by probabilistic tractography, and their WM integrity disruptions were found to be positively associated with the cognitive deficits in the FES patients.

**Discussion:** Preserved WM integrity may be a risk factor for suicidal ideation in FES patients. One possible explanation is that it contributes to preserved cognitive function, especially in working memory and verbal comprehension, which may be associated with greater insight and could lead to increased depression and suicidal ideation. The posterior corona radiata and the precuneus may be linked to the related biological processes.

## Introduction

Suicide is a complex process ranging from suicidal ideation to planning, attempting and committing suicide (1). Committing suicide is one of the major causes of death in schizophrenia (2). It was reported that approximately 20–40% schizophrenia patients attempt and 5.6% commit suicide in their lifetime (2–5). Identifying the characteristics of schizophrenia patients with suicidality is important for determining who is at the highest risk and should receive intensive treatment to prevent suicide (6).

Several functional and structural neuroimaging studies have attempted to find possible associations between brain characteristics and suicidality in schizophrenia, referring to both brain gray matter and white matter (WM) (7–11). Diffusion tensor imaging (DTI) offers a powerful tool to assess the microstructural integrity of brain WM (12). Factional anisotropy (FA) is often used as a measure of brain WM integrity which is thought to reflect fiber density, axonal diameter, and myelination in WM. A widespread increase in FA, indicating greater WM integrity, has been demonstrated in schizophrenia patients with suicide attempt history compared with those without such history (13). Suicidal ideation is the first step on the path to suicide (14), and we hypothesize that there may be similar associations between WM integrity and suicidal ideation. However, to our knowledge, no study has specifically focused on the associations between WM integrity and suicidal ideation in schizophrenia.

Cognitive deficit is considered a core feature of schizophrenia (15). Intriguing, several studies reported that poorer cognitive function is a possible protective factor of suicide in schizophrenia (6, 16–19), while no correlation between cognitive function and suicidality was observed in schizophrenia in some other studies (20–22). The inconsistency of previous data may be attributed to the difference in suicidality definition (i.e., suicidal ideation, suicide attempt, completed suicide or a combination of them) and cognitive assessment instruments (20). Besides, the WM integrity disruption was thought to be a primary contributor to several cognitive deficits, such as impaired processing speed and working memory (23). Alterations in WM integrity, therefore, would lead to related differences in cognitive function. However, what kind of roles the WM integrity alteration might play in the associations among itself, cognitive deficit and suicidality has never been investigated in schizophrenia patients so far.

The present study, for the first time, aimed to investigate the possible associations among suicidal ideation, WM integrity and cognitive functions in first-episode schizophrenia (FES) since the risk of suicide in schizophrenia is particularly higher during the first episode (16). At the same time, the potential confounding factors such as long-term medication, hospitalization and social isolation would be excluded (24). In addition, the schizophrenia patients with only suicidal ideation, those with a history of single suicide attempt and of multiple suicide attempts were reported to have different neurocognitive ability, suggesting that they are likely to be influenced by different neurocognitive factors (17). Therefore, a history of suicide attempt could also be a possible confounding factor and we excluded the FES patients with previous suicide attempts. We hypothesized that the FES patients with suicidal ideation may have greater WM integrity and better cognitive function, relative to those without suicidal ideation.

## Materials and methods

### Participants

Using the Structured Clinical Interview for DSM-IV Axis I Disorders, Patient Edition (SCID-I/P) (25), 63 FES patients, who were diagnosed with schizophrenia in the past 18 months without any previous episode of psychosis, were recruited from the Department of Psychiatry, the Second Xiangya Hospital of Central South University, Changsha, China. Forty-four age-, gender-, and education-matched healthy controls (HC) without family history of schizophrenia were also recruited for comparison. All participants met the following inclusion criteria: (a) 18–45 years of age; (b) Han Chinese ethnicity; (c) right handedness; (d) 9 years of education or above. Participants were excluded when they had: (a) a history of suicide attempt; (b) a history of any substance abuse; (c) a history of neurological disorders or severe physical diseases; (d) any contraindication to MRI; (e) a history of electroconvulsive therapy.

This study was approved by the Ethics Committee of the Second Xiangya Hospital of Central South University. All participants or guardians provided written informed signed consent for participation in the study.

### Measurements

Suicidal ideation was measured by the suicide item of the Calgary Depression Scale for Schizophrenia (CDSS), which was used to evaluate suicidal ideation in the past 2 weeks. The CDSS is a well-validated 9-item scale to assess depression in schizophrenia, with the scores for each item ranging from 0 to 3 (26). Using a single suicide item from depression scales to assess suicidal ideation has been proved to be valid (27). Hence, as previous researchers did (19, 28), FES patients were divided into two groups: one with current suicidal ideation (CDSS suicide item score > 0, SI+); the other one without current suicidal ideation (CDSS suicide item score = 0, SI–). Meanwhile, the subtotal of the remaining 8 of the 9 items of the CDSS (depression score), was used to reflect their depression symptom severity in the past 2 weeks (28).

A letter 2-back task as previously described (11) was conducted to measure working memory, a well-documented impaired cognitive function in schizophrenia (29), as it was reported to be related to suicidal ideation in schizophrenia in one previous study (17). Briefly, letters were displayed at the center of a screen, each for 500 ms followed by a 1,500 ms delay with a symbol “+” also at the center of the screen. Participants were then instructed to press the right button when the current letter was identical to what was displayed two trials before (target) and the left button when it was not (non-target). Each participant's mean reaction time and accuracy during the task were calculated. All participants also completed the Information and Digit Symbol subtests of Wechsler Adult Intelligence Scale-Chinese Revised (WAIS-CR) (30) to respectively measure verbal comprehension and processing speed (24).

In addition, all FES patients' current daily dosages of antipsychotics were recorded and converted to chlorpromazine equivalence (31), and the severity of their negative and positive symptoms were assessed by the Scale for the Assessment of Negative Symptoms (SANS) (32) and the Scale for Assessment of Positive Symptoms (SAPS) (33), respectively.

### Data acquisition and preprocessing

Diffusion-weighted and T1-weighted images were obtained on a 3.0 T MRI scanner (Philips Achieva XT). During the image acquisition, all participants were asked to close their eyes and tried their best to keep steady. The diffusion-weighted images were acquired using an Echo Planar imaging sequence (one *b* = 0 and 32 directions with *b* = 1,000 s/mm^2^; repetition time = 6,590 ms; echo time = 70 ms; 60 slices, 144 × 144 matrix; field of view = 240 × 240 mm^2^, slice thickness = 2.5 mm) and high-resolution T1-weighted images were acquired using T1-weighted 3D turbo field echo (repetition time = 7.5 ms; echo time = 3.7 ms; flip angle = 8°; 180 slices, 256 × 200 matrix; field of view = 240 × 240 mm^2^, slice thickness = 1 mm), which was adapted to subsequent analysis.

Data preprocessing was performed using the program of FSL version 5.0.8 (www.fmrib.ox.ac.uk/fsl) (34). The diffusion-weighted images were firstly corrected for head motion and eddy current using an affine registration to the *b* = 0 image. Then non-brain tissues in diffusion-weighted and T1-weighted images were removed using brain extraction (BET) tool (35). After that, diffusion-weighted images were coregistered to T1-weighted images using FLIRT tool. Finally, DTIFIT tool was used to obtain the images of fractional anisotropy (FA) and mean diffusivity (MD), the two most commonly used parameters in DTI (36).

### Tract-based spatial statistics (TBSS)

Voxelwise group comparison of diffusion-weighted images was done by using TBSS version 1.2 in FSL (37). All subjects' FA images were first aligned to FMRIB58_FA standard-space image using a nonlinear registration and then used to create a mean FA skeleton representing the centers of all tracts common to all subjects (threshold of 0.2). After that, each subject's aligned FA and MD data were projected onto this skeleton and fed into voxelwise comparison.

Group comparison on the mean FA skeleton was performed in the framework of the general linear model (GLM) using FSL's Randomize tool (38). All analyses were conducted with 10,000 permutations and with age, gender as well as education level used as covariates. A one-way analysis of covariance (ANCOVA) *F*-test with group as factor (three levels: SI+, SI–, and HC) was performed followed by six *post-hoc t*-test comparisons (HC > SI+, SI+ > HC, HC > SI–, SI– > HC, SI+ > SI–, SI– > SI+) assessing between-group differences. *Post-hoc t*-tests results were identified within the boundaries of the *F*-test results. Significance was tested at *p* < 0.05 with threshold-free cluster enhancement (TFCE) correction (39) and family-wise error rate (FWE) correction for multiple comparisons. The WM tracts where FA or MD showed significant differences (>30 significant voxels) were labeled by the JHU ICBM-DTI-81 White-Matter Labels atlas (40) and the JHU white-matter tractography atlas (41), and enlarged using the TBSS_fill toolbox for illustrative purposes.

### Definition of ROIs and probabilistic tractography

According to TBSS analysis, the overlap of FA and MD differences between SI+ and SI– were defined as regions of interest (ROIs). From each ROI, a binary mask was created to extract FA/MD values and used as a seed mask for probabilistic tractography using BEDPOSTx and PROBTRACKx software from the FSL (42). Fiber tracking was performed from all voxels within each seed mask (25000 streamline samples per voxel, 0.5 mm step lengths, curvature threshold = 0.2) in native space. The results of the probabilistic tractography for each subject were divided by the “waytotal” for controlling tract size, thresholded at 1% (43), binarized, and transformed to standard space. Next, they were summed to produce group probability maps, and thresholded to display paths presented in at least one-third of subjects in each group (44).

### Non-voxel-based comparisons and correlations

All the non-voxel-based statistical analyses were performed using the SPSS 22.0 software. The demographic characteristics and cognitive variables among the three groups were compared using Chi-square test or one-way analysis of variance (ANOVA) followed by Tukey HSD *post-hoc* tests. The clinical characteristics were compared between the SI+ and SI– groups using two-sample *t*-tests.

Mean FA and MD values were calculated from each ROI using the fslmeants tool. To exam their associations with the cognitive performances and the depression score in the FES patients, partial correlation analysis was then performed after controlling for age, gender, years of education and SAPS/SANS scores. The partial correlations were also computed between the FA/MD values and SAPS/SANS scores after controlling for age, gender and years of education.

## Results

### Demographic and clinical characteristics

Eighteen patients (18/63, 28.6%) reported current suicidal ideation. Among FES patients with (SI+, *n* = 18)/without (SI–, *n* = 45) current suicidal ideation and healthy controls (HC, *n* = 44), no significant between-group differences were observed in age, gender, education level, SAPS/SANS total scores, course of disease or the dosage of antipsychotics. Higher depression scores (*t* = −2.506, *p* = 0.015) were observed in the group of SI+ compared to SI– (Table 1).

**Table 1:** The demographic and clinical characteristics of the patients without suicidal ideation (SI–), the patients with suicidal ideation (SI+) and healthy controls (HC).

	**SI− (*n* = 45)**	**SI+ (*n* = 18)**	**HC (*n* = 44)**	**Group comparisons**
	**(Mean ± SD)**	**(Mean ± SD)**	**(Mean ± SD)**	
Age (years)	24.58 ± 5.93	24.83 ± 6.91	23.20 ± 6.49	*F* = 0.687, *p* = 0.505
Gender (male/female)	25/20	9/9	21/23	χ^2^ = 0.563, *p* = 0.755
Education (years)	11.80 ± 2.39	11.75 ± 1.73	12.59 ± 1.69	*F* = 2.173, *p* = 0.119
Course of disease (months)	8.50 ± 5.01	6.44 ± 4.74	/	*t* = 1.494, *p* = 0.140
Chlorpromazine equivalents (mg/day)	241.29 ± 153.33	222.72 ± 130.20	/	*t* = 0.452, *p* = 0.653
SAPS score	21.42 ± 17.07	24.56 ± 11.59	/	*t* = −0.714, *p* = 0.478
SANS score	33.11 ± 27.35	34.72 ± 21.82	/	*t* = −0.233, *p* = 0.824
Depression score	2.91 ± 3.07	5.28 ± 4.08	/	*t* = −2.506, *p* = 0.015

### Cognitive performances

Among the three groups, significant differences were observed in the performances of WAIS-Information (WAIS-I) test, WAIS-Digit Symbol (WAIS-DS) test as well as target-accuracy and target-reaction time (RT) in the 2-back task (Table 2). Using Tukey HSD *post-hoc* analysis, the SI– patients were found to have lower WAIS-I score (*p* = 0.001), lower WAIS-DS score (*p* < 0.001), lower 2-back target-accuracy (*p* < 0.001) and longer 2-back target-RT (*p* = 0.019) compared with HC. The SI+ patients were also found to have a lower WAIS-DS score than HC (*p* < 0.001). However, no significant differences were observed between the SI+ patients and HC in the performances of WAIS-I (*p* = 0.591), 2-back target-accuracy (*p* = 0.082) or 2-back target-RT (*p* = 0.559).

**Table 2:** The between-group comparisons on cognitive performances among the patients without suicidal ideation (SI–), the patients with suicidal ideation (SI+) and healthy controls (HC) using one-way analysis of variance (ANOVA) followed by Tukey HSD *post-hoc* tests.

	**SI− (*n* = 45)**	**SI+ (*n* = 18)**	**HC (*n* = 44)**	**ANOVA**	**Tukey**
	**(Mean ± SD)**	**(Mean ± SD)**	**(Mean ± SD)**		
WAIS-I score	15.47 ± 4.22	17.89 ± 5.82	19.15 ± 4.41	*F* = 7.271, *p* = 0.001	SI– < HC
WAIS-DS score	62.04 ± 14.08	66.06 ± 15.65	85.25 ± 10.28	*F* = 38.219, *p* < 0.001	SI–, SI+ < HC
2-back target-accuracy (%)	46.80 ± 24.91	55.89 ± 26.11	69.90 ± 20.29	*F* = 11.170, *p* < 0.001	SI– < HC
2-back target-RT (ms)	725.48 ± 199.40	675.70 ± 131.28	627.01 ± 146.34	*F* = 3.785, *p* = 0.026	SI– > HC
2-back non-target-accuracy (%)	79.38 ± 20.59	77.83 ± 27.50	84.72 ± 16.61	*F* = 1.078, *p* = 0.344	
2-back non-target-RT (ms)	664.39 ± 191.59	650.12 ± 204.89	634.06 ± 144.07	*F* = 0.331, *p* = 0.719	

### TBSS and probabilistic tractography

**Figure 1:**
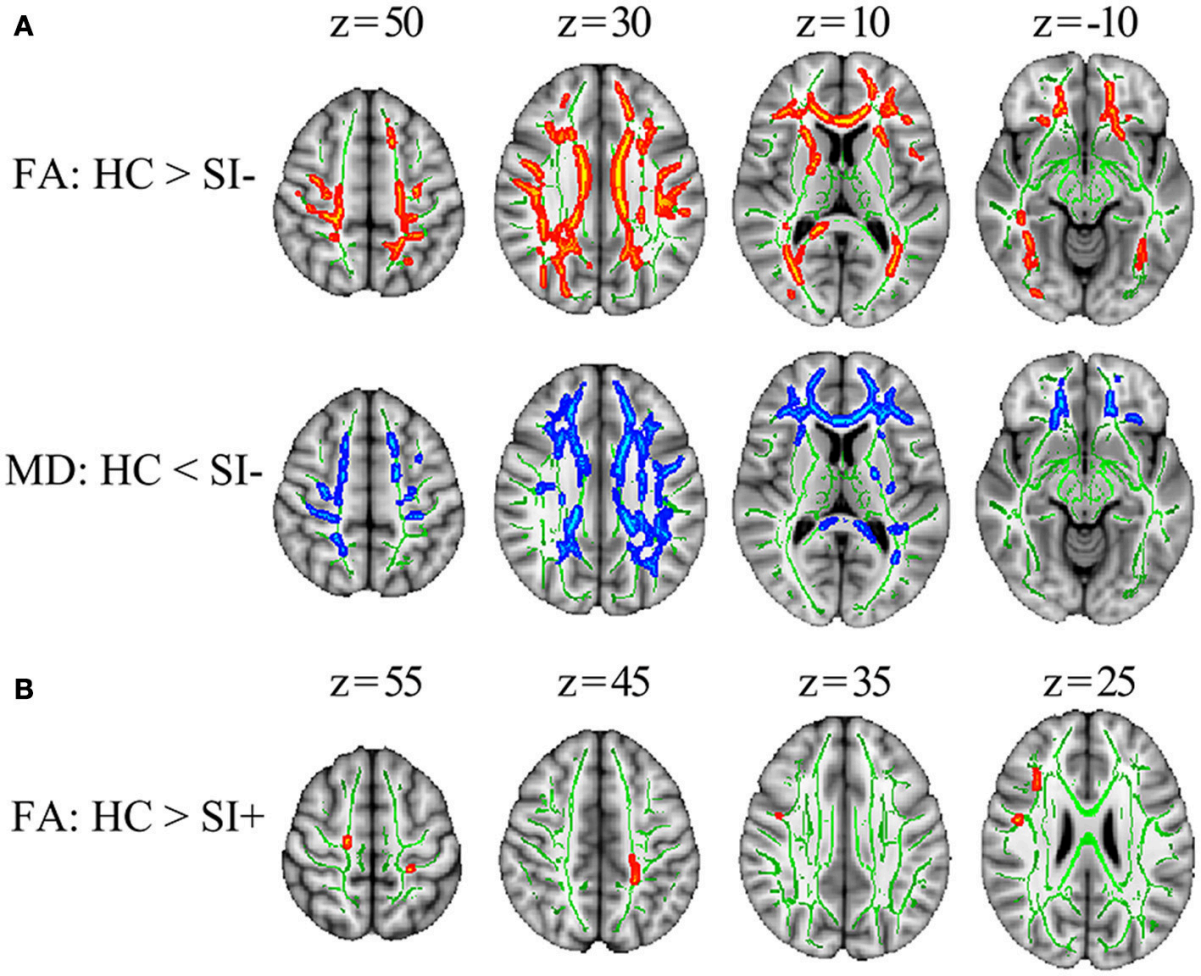
The TBSS results comparing FES patients and healthy controls (HC). **(A)** The differences in FA and MD between the patients without suicidal ideation (SI–) and HC. **(B)** The differences in FA between the patients with suicidal ideation (SI+) and HC. Significant areas (*p* < 0.05, corrected) were marked in orange (higher in HC) or blue (lower in HC) with underlying WM skeleton marked in green. FA, fractional anisotropy; MD, mean diffusivity; x, y, z, MNI coordinates.

Compared with HC, the SI– patients showed decreased FA in multiple WM tracts including corpus callosum (genu, body, and splenium), bilateral corona radiata (anterior, superior, and posterior), bilateral anterior limb of internal capsule, bilateral posterior thalamic radiation (include optic radiation), left external capsule, bilateral superior longitudinal fasciculus, bilateral superior fronto-occipital fasciculus and right sagittal stratum (*p* < 0.05, corrected) as well as increased MD in corpus callosum (genu, body, and splenium), bilateral corona radiata (anterior, superior, and posterior), left anterior limb of internal capsule, left posterior limb of internal capsule, left posterior thalamic radiation (include optic radiation), left external capsule and bilateral superior longitudinal fasciculus (*p* < 0.05, corrected) (Figure 1A). Compared with HC, the SI+ patients showed decreased FA in bilateral anterior thalamic radiation, bilateral corticospinal tract, right inferior fronto-occipital fasciculus and bilateral superior longitudinal fasciculus (*p* < 0.05, corrected) but no difference in MD (*p* > 0.05, corrected) (Figure 1B). No region showed increased FA or decreased MD in the FES patients compared to HC (*p* > 0.05, corrected).

**Figure 2:**
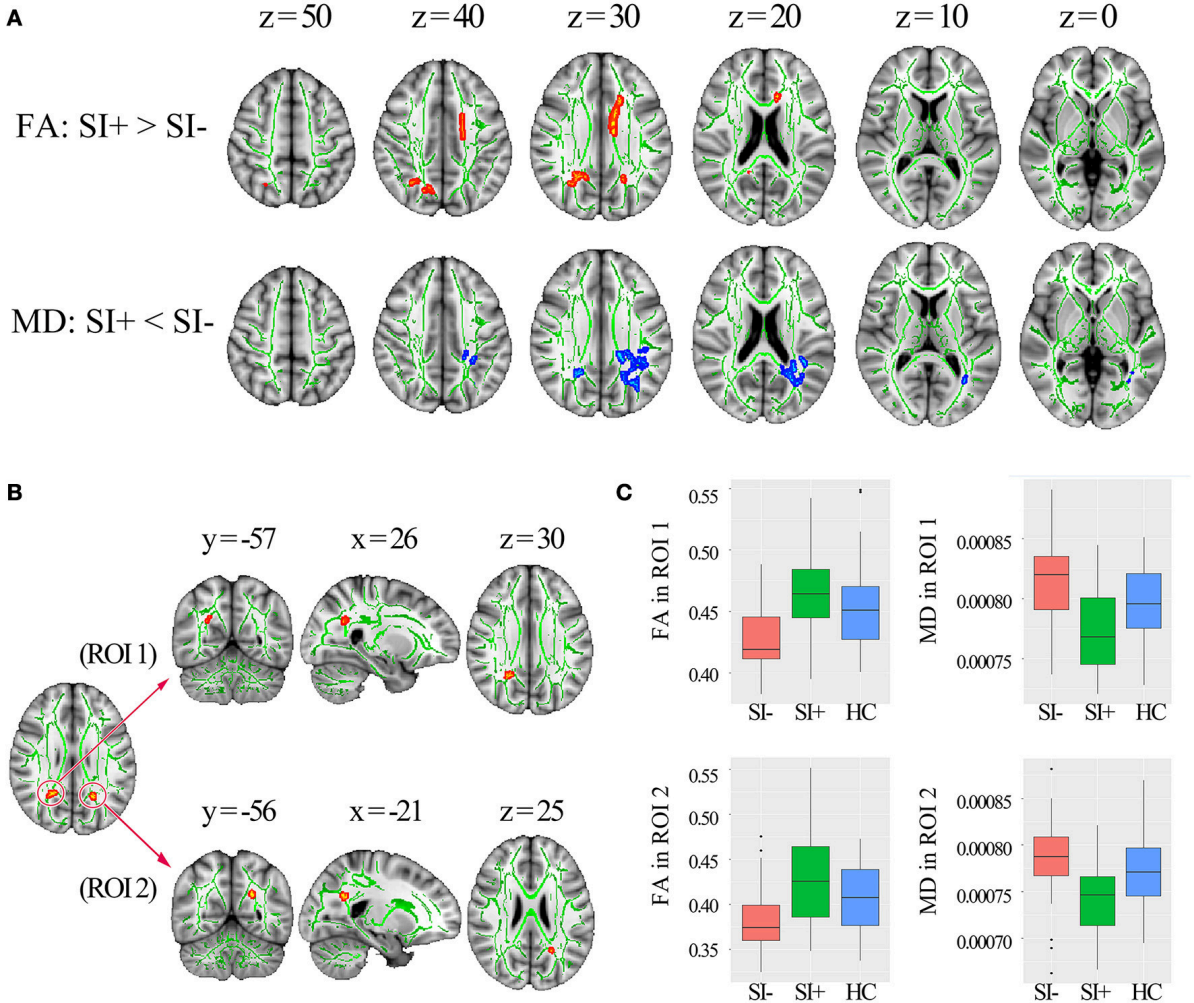
The TBSS results comparing the FES patients with (SI+) and without (SI–) suicidal ideation. **(A)** The differences in FA and MD (*p* < 0.05, corrected) between SI+ and SI–, marked in orange (higher in SI+) or blue (lower in SI–) with underlying WM skeleton marked in green. **(B)** Two regions of interest (ROI) located in right and left posterior corona radiata identified by overlapping the brain regions with FA and MD differences between SI+ and SI. **(C)** Mean FA and MD values extracted from right posterior corona radiata (ROI 1) and left posterior corona radiata (ROI 2) in the groups of SI+, SI–, and healthy controls (HC) respectively. FA, fractional anisotropy; MD, mean diffusivity; x, y, z, MNI coordinates.

Compared with SI–, the SI+ patients showed higher FA in corpus callosum (genu, body, and splenium), left anterior corona radiata, left superior corona radiata and bilateral posterior corona radiata (*p* < 0.05, corrected) as well as lower MD in splenium of corpus callosum, bilateral posterior corona radiata, left posterior thalamic radiation and left superior longitudinal fasciculus (*p* < 0.05, corrected) (Figure 2A). No region showed lower FA or higher MD in SI+ compared to SI– (*p* > 0.05, corrected). The overlap of FA and MD differences between SI- and SI+ was shown in Figure 2B, including one 122-voxel cluster in the right posterior corona radiata (defined as ROI 1) and one 44-voxel cluster in the left posterior corona radiata (defined as ROI 2). The FA and MD values were extracted from each ROI for following correlation analysis, with mean values in each group shown in Figure 2C.

**Figure 3:**
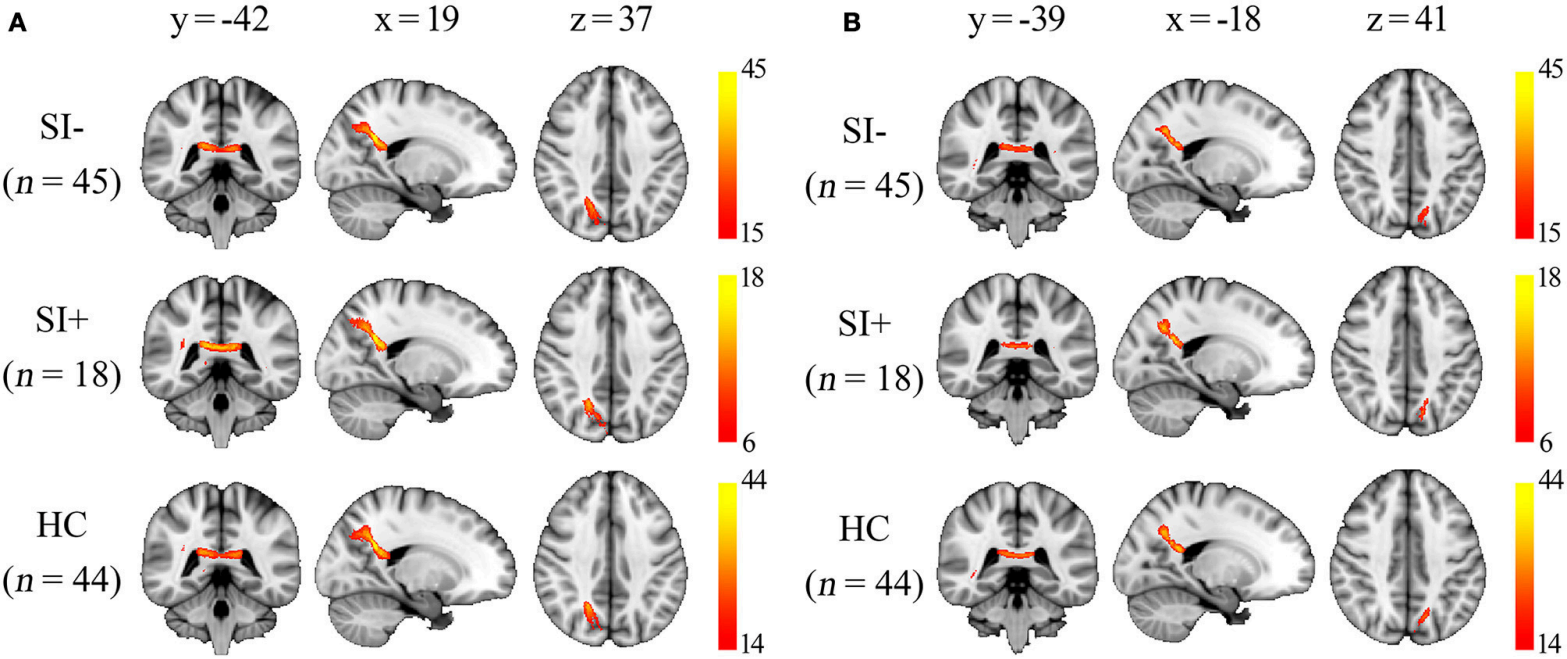
The group probability maps generated from the two regions of interest (ROI) as described in Figure 2B. The maps were thresholded to display the paths presented in at least one-third of subjects in each group, and the color bars represent the proportion of subjects with connection to the ROI in each voxel. **(A)** Maps generated from the ROI 1 in right posterior corona radiata. **(B)** Maps generated from the ROI 2 in left posterior corona radiata. x, y, z, MNI coordinates.

Using separate probabilistic tractography analysis, similar tract profiles were obtained from the three groups (SI–, SI+, HC). The right posterior corona radiata (ROI 1) was connected to right precuneus and the left posterior corona radiata (ROI 2) was connected to left precuneus. Meanwhile, they were connected to each other through the corpus callosum (Figure 3).

### Correlation analysis

**Figure 4:**
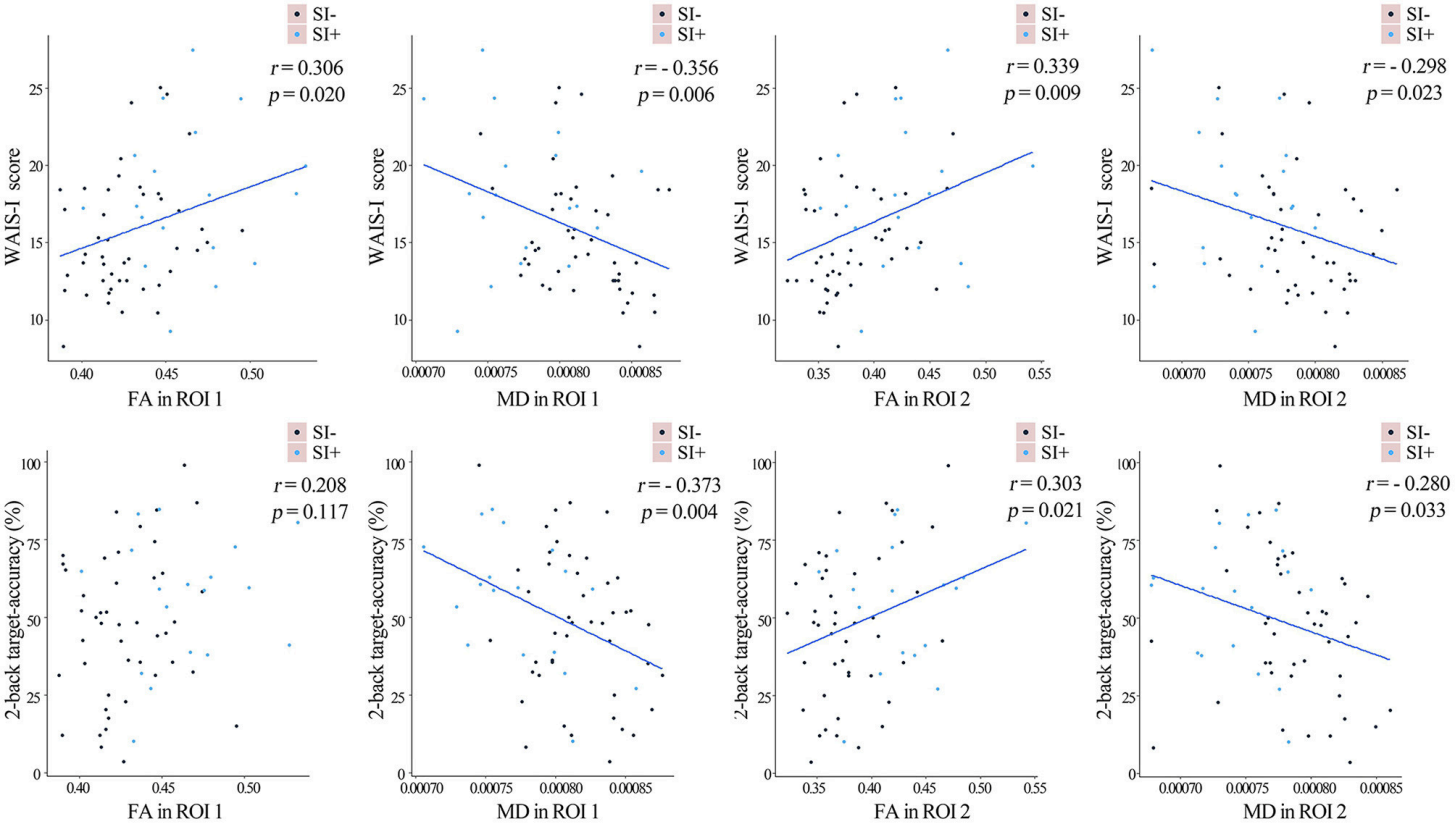
The linear fits representing the partial correlations between the FA/MD values in the two regions of interest (ROI) as described in Figure 2B and cognitive performances in the whole sample of FES patients, after controlling for age, gender, years of education and SAPS/SANS scores. The FES patients with (SI+) and without (SI–) suicidal ideation were marked in blue and purple separately. FA, fractional anisotropy; MD, mean diffusivity.

In the patients, FA in ROI 1 (*r* = 0.306, *p* = 0.020), MD in ROI 1 (*r* = −0.356, *p* = 0.006), FA in ROI 2 (*r* = 0.338, *p* = 0.009) and MD in ROI 2 (*r* = −0.298, *p* = 0.023) were all found to significantly correlate with the WAIS-I score. MD in ROI 1 (*r* = −0.373, *p* = 0.004), FA in ROI 2 (*r* = 0.303, *p* = 0.021) and MD in ROI 2 (*r* = −0.280, *p* = 0.033) were found to significantly correlate with the 2-back target-accuracy (all uncorrected for multiple comparisons, as shown in Figure 4). No significant correlations were found between FA/MD values and the depression score, or between FA/MD values and the SAP/SANS scores.

## Discussion

In the present study, we found that compared with the FES patients without current suicidal ideation (SI–), the patients with current suicidal ideation (SI+) showed (1) greater WM integrity, indicated by significantly higher FA or lower MD in multiple WM tracts and specifically, higher FA coupled with lower MD in two clusters located in bilateral posterior corona radiata; (2) less cognitive deficit in working memory and verbal comprehension. Furthermore, the FA and MD values in bilateral posterior corona radiata were found to be correlated with the patients' cognitive performances of working memory and verbal comprehension.

The first important finding of the present study is that we observed greater WM integrity in SI+ than SI–. FA and MD describe complementary information that FA reflects the membrane, myelin and fiber integrities while MD is mainly related to the tissue integrity (45–47). Compared to HC, widespread decreased FA and increased MD were observed in SI− (Figure 1A), which may reflect an extensive WM integrity disruption including axonal loss and myelin degeneration in these WM tracts (48). In SI+ compared with HC, however, decreased FA was observed in only a few WM tracts and no significant MD increase was observed (Figure 1B). The isolated FA decrease without MD increase may be attributable to a relative mild WM change (48). In summary, the WM integrity disruption, which has been widely reported in FES (49–51), was observed in both the SI– and SI+ patients; but the WM disruption in SI+ was found to be milder and more localized. Compared with SI−, significantly higher FA or lower MD was also observed in multiple WM tracts in SI+ (Figure 2A), indicating greater WM integrities in these regions. Specifically, higher FA coupled with lower MD were observed in bilateral posterior corona radiata (Figure 2B). These results suggest that less disruption of WM integrity, especially in posterior corona radiata, may be associated with suicidal ideation in FES. Our findings, for the first time, demonstrate a possible association between the WM integrity alterations and suicidal ideation in schizophrenia.

The second major finding of the present study is that we observed less cognitive deficit in working memory and verbal comprehension in SI+ than SI−. The cognitive deficit in schizophrenia patients is well documented and seven domains were highlighted by previous investigators: processing speed, attention/vigilance, working memory, verbal learning and memory, visual learning and memory, reasoning and problem solving, and verbal comprehension (52). However, only a limited number of studies have investigated the relationship between cognitive deficit and suicidality in schizophrenia and reported inconsistent results (6, 17, 18, 20–22). Verbal comprehension, processing speed and working memory were assessed by WAIS-I, WAIS-DS, and 2-back tasks in the present study. In SI–, we found significant impairments in all the three domains. In SI+, however, only significantly impaired processing speed was observed. As shown in Table 2, the performances of SI+ patients in WAIS-I and 2-back tasks, which assess verbal comprehension and working memory, were between SI– and HC and showed significant difference with neither of them. These results imply that less cognitive deficit in working memory and verbal comprehension may be related to suicidal ideation in schizophrenia. The result was similar to one previous study conducted by Delaney et al. (17), which reported that schizophrenia patients with suicidal ideation or single attempt had better cognitive functions including working memory than the non-suicidal patients.

In schizophrenia, both better working memory and better verbal comprehension were ever reported to be associated with greater insight, which is defined as the awareness of having a mental disorder and need for treatments (16, 53, 54). It was thought that more insight into the disorder could contribute to increased hopelessness and depression, then leading to suicide (2). In fact, an association between greater insight and increased risk of suicide in first episode psychosis has been widely reported (55–57). Although the insight was not evaluated, the SI+ group were indeed more depressive than SI– in the present study (Table 1). These might partly interpret why less cognitive deficit in working memory and verbal comprehension was found to be associated with suicidal ideation in the present study.

In order to further investigate the relationship between WM integrity disruption and cognitive deficit in FES patients, the partial correlations were assessed after controlling potential confounding factors including age, gender, years of education and SAPS/SANS scores. The FA and MD values in bilateral posterior corona radiata were found to significantly correlate with the 2-back target-accuracy and WAIS-I score, which assess working memory and verbal comprehension (Figure 4). No significant correlations were found between FA/MD values and clinical symptoms. The effects of some other factors, such as long-term medication and a history of electroconvulsive therapy, were also excluded by including only the first-episode patients without such a history. The results suggested that preserved WM integrity in bilateral posterior corona radiata is associated with preserved working memory and verbal comprehension, which were consistent with the previous findings that WM integrity disruption may be a primary contributor to several cognitive deficits in schizophrenia (23). Therefore, as discussed previously, one possible explanation why preserved WM integrity was found to be related to higher possibility of having suicidal ideation in FES patients is that it contributes to preserved cognitive function, which is associated with greater insight and may lead to increased depression and suicidal ideation.

The corona radiata is a bundle of projection fibers that carries almost all of the neural traffic from and to the cerebral cortex (58). Decreased FA in posterior corona radiata has been reported in a meta-analysis comparing mild cognitive impairment patients to healthy controls (59), and also found to correlate with the cognitive deficit in several different diseases (60–62). However, how the WM integrity in corona radiata impacts cognitive function is unclear. The WM tracts of the bilateral posterior corona radiata, where significantly higher FA coupled with lower MD were observed in SI+ compared with SI–, contain fibers mainly to bilateral precuneus and also fibers connecting the hemispheres (Figure 3). Precuneus was thought to be related to a wide range of higher-order cognitive functions (63). The precuneus also plays a pivotal role in the default mode network (DMN), which is composed of a set of brain regions including the precuneus, medial prefrontal cortex (mPFC), posterior cingulate cortex (PCC) and lateral/medial temporal lobes (64). It has been proved that the functional connectivity within the entire DMN is based on distinct pattern of WM microstructure (65). Therefore, we assumed that disrupted WM integrity in the posterior corona radiata could possibly interfere with the normal communication between bilateral precuneus and lead to the disruption of DMN activity. The DMN is typically active “by default” when the brain is at wakeful rest, but suppressed during certain goal-oriented tasks such as a working memory task (66). Disrupted DMN activity, which is expressed as inefficient DMN suppression, has been proved to play a specific neuro-pathological role in the cognitive deficits in FES (24). Interestingly, it was found that less DMN suppression was also associated with poorer insight in schizophrenia spectrum disorders (67). These results suggest that less disruption of DMN activity may lead to preserved cognitive function, greater insight, and then higher possibility of having suicide ideation as we discussed earlier. In fact, there were more evidences that support our assumption. For example, the hypo-connectivity from mPFC to PCC, two important regions within the DMN, was found to represent a possible correlate of increased suicide risk in FES (11). Moreover, the association between abnormal DMN activity and suicidality has been proved in depressed adolescents (68). Nevertheless, there has been no study to investigate the association between the DMN activity and suicidality in schizophrenia to our best knowledge, but it is worth looking into in the future.

In summary, we concluded that preserved WM integrity may be a risk factor of suicidal ideation in FES patients. One possible explanation is that it contributes to preserved cognitive function, especially in working memory and verbal comprehension, which may be associated with greater insight and then could lead to increased depression and suicidal ideation. The posterior corona radiata and the precuneus may be linked to the related biological processes. Our findings will contribute to a better understanding of the neuropathophysiological basis of suicidal ideation in schizophrenia.

Our study has various limitations. First, the sample size was small, particularly in the patients with suicidal ideation (*n* = 18). Second, the present study only included three cognitive domains: working memory, verbal comprehension and processing speed, but not any other cognitive domain or the full-scale intelligence quotient. Third, the insight, which was considered to play an important role in the associations among WM integrity, cognitive function and suicidal ideation in schizophrenia as discussed, was not examined in the present study and should be included in the future studies. Furthermore, we only investigated the role of WM microstructural integrity in the process of suicide and its relationship with cognitive deficits. In the future, multimodal studies that integrate structural (WM and gray matter) and functional (resting-state and task-based) imaging are necessary to further understand the neuropathophysiological basis of suicidal ideation in schizophrenia and find the possible underlying alterations in neural networks.

## Author contributions

YL and XO designed the study and supervised the study. YL, XO, XC, and ZL collected the imaging data and clinical information. YL, WP, and BS carried out the analysis. YL wrote the first draft of manuscript. ZL, XH, EL, EC and RR contributed to the final manuscript.

### Conflict of interest statement

The authors declare that the research was conducted in the absence of any commercial or financial relationships that could be construed as a potential conflict of interest.
